# Building cooperation through health initiatives: an Arab and Israeli case study

**DOI:** 10.1186/1752-1505-1-8

**Published:** 2007-07-17

**Authors:** Harvey A Skinner, Abi Sriharan

**Affiliations:** 1 Dean, Faculty of Health, York University, 4700 Keele Street, Toronto, ON, M3J 1P3, Canada; 2 Canada International Scientific Exchange Program (CISEPO), Toronto, Canada; 3 Deputy Director, Peter A. Silverman Centre for International Health, Mount Sinai Hospital, 600 University Ave, Toronto, M5G 1X5, Canada

## Abstract

**Background:**

Ongoing conflict in the Middle East poses a major threat to health and security. A project screening Arab and Israeli newborns for hearing loss provided an opportunity to evaluate ways for building cooperation. The aims of this study were to: a) examine what attracted Israeli, Jordanian and Palestinian participants to the project, b) describe challenges they faced, and c) draw lessons learned for guiding cross-border health initiatives.

**Methods:**

A case study method was used involving 12 key informants stratified by country (3 Israeli, 3 Jordanian, 3 Palestinian, 3 Canadian). In-depth interviews were tape-recorded, transcribed and analyzed using an inductive qualitative approach to derive key themes.

**Results:**

Major reasons for getting involved included: concern over an important health problem, curiosity about neighbors and opportunities for professional advancement. Participants were attracted to prospects for opening the dialogue, building relationships and facilitating cooperation in the region. The political situation was a major challenge that delayed implementation of the project and placed participants under social pressure. Among lessons learned, fostering personal relationships was viewed as critical for success of this initiative.

**Conclusion:**

Arab and Israeli health professionals were prepared to get involved for two types of reasons: a) Project Level: opportunity to address a significant health issue (e.g. congenital hearing loss) while enhancing their professional careers, and b) Meta Level: concern about taking positive steps for building cooperation in the region. We invite discussion about roles that health professionals can play in building "cooperation networks" for underpinning health security, conflict resolution and global health promotion.

## Background

"When I went to Nicosia, I was like, why am I going? I mean, what's going to happen? I mean, why is anybody even bothering? Do these people still believe in such things? I have lots of questions going on and when we reached there and we talked, it was like being in a dream, you know. You see Israelis that are willing still to help Palestinians, I am seeing Palestinians that are still willing to hear Israelis". (P6)

Participant at the first CISEPO Middle East international research conference, Nicosia, Cyprus, October 23–24, 2002.

As this quotation illustrates, bringing people together from a conflicted region to work on a common initiative can have a transformative impact. The Middle East provides a complex environment for studying initiatives aimed at using health as a bridge for peacebuilding [1-4]. The Israeli and Palestinian conflict, in particular, embodies a fault line of contested history, disputed entitlements and power differentials. Why would health professionals step forward to collaborate in such a conflicted zone? What barriers need to be addressed in moving from talk to action? How can the politics and inevitable 'hotspots' be managed successfully?

Some argue that successful initiatives should start only after a political resolution[5]. There is a pressing need for evidence, not ideology. To date, there has been little research to understand how to move peace through health initiatives successfully from concept to implementation [6], stimulating calls for a new discipline [7,8].

Cross sectional surveys have shown a significant relationship between health professionals' involvement in inter-ethnic activities and their willingness to collaborate [6]. However, there has been little systematic evaluation to understand whether this is a causal relationship. Yusef and colleagues [9] describe war as a disease process that can be prevented from developing or modified, and if required can be treated or rehabilitated. It is important to put in place necessary measures to prevent future outbreaks from developing. However, others such as Jabbour [5] argue that successful "peace-through-health" initiatives start only after the political resolution. If that's the case, then are there constructive steps health professionals can take until political leaders work out the conflict?

For the past decade, Israeli, Jordanian and Palestinian colleagues have worked together through the Canada International Scientific Exchange Program[10,11]. In May 1998, the *Middle East Association for Managing Hearing Loss *(*MEHA*) was established as the first joint Arab and Israeli professional association. Its aim is to promote cooperation by advancing knowledge and services related to congenital hearing impairment [12] – a priority iss ue for the region due to high rates of consanguinity ranging from 36–50% in Jordan up to 53% among certain Arab communities in Israel [13,14]. MEHA's first study (Project 1) screened and habilitated 17,000 Jordanian, Palestinian and Israeli newborns during April 2001–June 2004 [15]. Initially planned as a two-year project, the timeline was extended due to difficulties in the region surrounding the second intifada (uprising of Palestinians after September 28, 2000).

**Figure 1:**
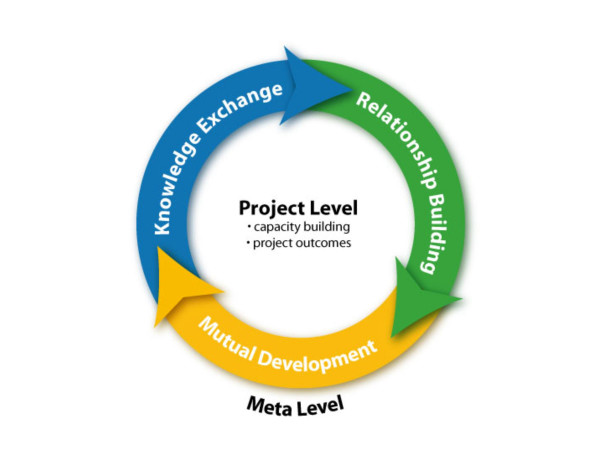
Bi-level model for peacebuilding.

The formation of MEHA and Project 1 provided an opportunity to study the dynamics of collaboration during a period of significant conflict. The aims of this study were to: a) examine what attracted Israeli, Jordanian and Palestinian health professionals to MEHA and its initial project, b) identify challenges and barriers faced, and c) draw lessons for a model to guide the building of cooperation networks through international health initiatives. This case study is guided by a model [10] for global health initiatives to achieve both project specific outcomes in health improvement and broader impact on knowledge exchange, mutual understanding and cooperation (Figure 1).

## Methods

### Approach

A case study method [16] was used involving key informant interviews to provide in-depth information regarding the dynamics of cross-border involvement of colleagues in a project screening Arab and Israeli newborns for hearing loss (MEHA Project 1). Guiding questions were developed, in part, using the research objectives as a framework:

1. How successful has MEHA Project 1 been in meeting its initial goals,

2. What do you see as the main factors contributing to its success,

3. What do you see as the main challenges (barriers),

4. Describe a particular incident/event that was critical to MEHA Project 1 success,

5. If you were to start again what would you do differently – what would you do the same,

6. What do you need to do to ensure future success,

7. How does MEHA project 1 contribute to broader goals of building relationships, cooperation and trust?

### Subjects

The sample included 12 key individuals (10 males; 2 females) who played various roles in the formation of MEHA and its initial project. The sampling frame stratified participants by country (3 Israeli, 3 Jordanian, 3 Palestinian, 3 Canadian) and health profession (physician, audiologist, MEHA steering committee member).

### Process

Interviews with the Canadian participants were face-to-face and the Middle East participants were by telephone. To help them prepare for the interview, a copy of the guiding questions was sent prior to the interview. All measures were taken to minimize the interviewer's role in the interview and to avoid leading questions. During the interviews, the participants were encouraged to give their opinion; the interviewer merely acted to trigger the thought process of the participant and then actively listened.

At the end of the interview participants were encouraged to share any additional comments. The same researcher (HAS) conducted all interviews which took for 45–60 minutes, and notes were taken by an independent observer (AS).

### Research Ethics

A study protocol was approved by the Human Subjects Ethics Review Committee, University of Toronto. Participation in the interview was on volunteer basis. All the participants signed a consent form. The consent form had two purposes: 1) to ensure confidentiality and anonymity of participants; 2) to inform them that their comments will be used for research purposes only. The two authors were not involved in MEHA or Project 1, but work on other areas of CISEPO which facilitated their entry and trust with the participants. This facilitated their entry and trust with the 12 key informants regarding MEHA project 1. Care was taken to ensure confidentiality and anonymity (e.g. no individual results were shared among the interviewees during or following the study).

### Data Analyses

To ensure trustworthiness of the analysis and interpretation [17], the interviews were conducted using a structured guide and themes were cross-checked independently. First, the interviews were tape-recorded and then professionally transcribed for data compilation. Then, key themes were identified by one researcher (AS) and independently checked by the second researcher (HAS). Comparisons were made on the basis of frequency (how often was it said), extensiveness (how many people said it), and intensity (how strong was the opinion). Finally, the coding framework and summary tables were reviewed for authenticity by the 12 interviewees (member check).

Emphasis in this manuscript is on presenting first order interpretations (descriptive level), rather than higher order interpretations connecting themes to deeper theoretical constructs [17].

## Results

### Reasons for involvement

Table 1 summarizes major reasons why participants got involved in this health initiative. At the project level (Figure 1), a key theme was the opportunity to address an important health issue (congenital hearing loss) of mutual concern for people in this region. The expectation was that involvement would lead to practical outcomes for screening and habilitating newborns for hearing loss in the region. A related reason involved an altruistic concern for helping needy children. Participants were interested in finding out how their neighbors were conceptualizing and organizing health and community services for hearing loss. Also, they saw opportunities for professional advancement through involvement in this network (e.g. research presentations, joint publications, promotion letters).

**Table 1:** Reasons for getting involved in cross-border activities

**I. Project Level**
**Important Mutual Problem**
*"Health is something common and it doesn't stop with any boundaries. Hearing loss is recognized as one of the epidemics, (I may use that word) in the region. It is common. It seems to be the most common congenital disorder in children." (P8)*
**Curiosity**
*"I am very, very interested in ....how they (neighboring states) treat deafness, what is the attitude towards deafness, and I'm still very surprised how they treat it." (P4)*
**Humanitarianism Concern**
*"I think it's very important if we can help, just help children. Not in the political basis, just as human beings." (P4)*
**Professional Advancement**
*"Networking opportunity with the international community, who have excelled in this field." (P3)*
**Trusted Third Party**
*"The role of Canada has been very positive in providing an umbrella for these activities, for these contacts. It really has been indispensable ... at the end of the day I think we would like to see Jordanians, Palestinians and Israelis coordinating, co-operating, co-existing, living together and working without that umbrella, but in the short term it has been needed." (P7)*

**II. Meta Level**

**Relationship Building**
*"... knowing from the other side someone may listen to you one day when you need them and that maybe things will be better and real cooperation can really come to life."(P6)*
**Mutual Development**
*"We don't have any alternative. I think at the end of the day we will be dealing with each other, living with each other and it's better to start now than to start much later...not only for the sake of peace building but for the sake of our children." (P7)*
**Knowledge Exchange**
*"... willingness of the people to take a chance, and to work with each other... and really want to do some problem solving and work together on research." (P1)*
**Personal Relationships**
*"I think once they saw the human side of each other, I think friendships grew and those friendships have been sustained over the difficult times." (P7)*

At a meta level (Figure 1), the main themes reflected a commitment to taking positive steps for cooperation and peace building. Participants expressed the need for being realistic and pragmatic – beginning with listening and dialogue: *"I really do believe that peace can be made only when people are speaking or talking." (P4) *A related theme involved mutual development, realizing that Arabs and Israelis are living with each other in the region. Knowledge exchange and capacity building were important tangible benefits. Also, the salience of personal relationships (humanizing effect) proved to be a powerful attractor for keeping participants involved during difficult periods.

### Challenges faced

The main issues are described in Table 2. The political situation in the region was a critical challenge. Participants recalled that the project was planned during 1998/1999 when the region was experiencing relative calm. Then, conflict heightened significantly during September 2000 with initiation of the second intifada. The impact included frequent delays and cancellation of meetings. Despite initial good will among colleagues regarding the project, considerable attention was needed to bolster morale and address burn out. Ongoing support and leadership from the Canadian participants were seen as vital at this early stage of the collaboration.

**Table 2:** Challenges of cross-border involvement

**Political Situation and Burnout**
*"Political pressures limit the extent to which MEHA can operate and then on a more individual basis ...People can easily wear out." (P1)*
**Social Pressure**
*"Collegial and societal pressures on partners mitigates against face-to-face meetings and other cooperative ventures." (P5)*
**Personal Exposure**
*"Some people are angry about this relationship, so it is very difficult to speak about the whole people here... So we have a great problem to expose ourselves, even to the professionals." (P4)*
**Achieving Practical Results**
*"Only few people saw the importance or the value of this project at the very beginning. One, because they didn't see any tangible results, as it were, on the ground...seeing is believing, and the centre which was opened in Amman by itself was something to me that was very moving... it lifted the morale of those who are involved." (P3)*
**Asymmetrical Distribution of Knowledge and Resources**
*"... academic level in Israel is much higher than in Jordan. It takes much more time to achieve the same level. But even so I think we have a great success." (P9)*
**Leadership**
*"Leadership has been very dynamic in keeping all parties involved, even at points when things are politically deteriorating." (P10)*
**Lack of Recognition**
*"Everybody knows what MSF is, but people don't quite know what CISEPO is." (P1)*
**Sustainability**
*"We need ... to train a younger generation of (leaders) for the long-term sustainability of CISEPO and MEHA over the next ten, twenty years... maintain what we have over the long run." (P7)*

A related challenge was maintaining connection among partners, although the advent of the Internet – especially email – greatly facilitated communication. The Canadian partners played an important role with frequent phone and email contact, visits to the region and organization of cross-boarder meetings and scientific events each year. Another challenge was managing the regional profile of the project. Participants described situations where they had to be careful when speaking about their involvement in cross-boarder activity due to concern over reactions by colleagues and institutions. Special efforts were needed to work 'below' the politics and manage media exposure in the region.

The asymmetrical distribution of scientific knowledge and resources was noted by participants. For example, hospital based resources in Israel and support of the Royal Medical Services in Jordan enabled both to achieve a target of 8,000 screened newborns, whereas a lower level of resources and restrictions in travel posed major barriers for screening newborns in the Palestinian territories (1,000 were successfully screened). Limited funding, exacerbated by having to extend the project timeline due to the conflict, put restrictions on running the project smoothly. Participants pointed out that MEHA and CISEPO have relatively low recognition and regional branding. Although this low profile helped minimize political and social pressure on participants, it worked against success in fundraising. This poses a significant challenge for sustaining the collaboration.

Due to political realities in the regions, participants also expressed feelings of ambivalence and caution about cross-border cooperation, especially when proximal to a traumatic event. This was expressed by one participant:

*"One has to bear in mind that while we want to assist and cooperate with our neighbors, we also don't want to, and we should not assist them in developing an institution which might have immediate political or harmful political implications.*"(P9)

### Lessons learned

A number of lessons can be gleaned from this initiative (Table 3). In looking back on the project, participants underscored success factors that would do the same: focus on a mutual health concern, engage like-minded professionals, keep a positive mental attitude, and treat partners equitably. The importance of moving from talk to action was emphasized. Tangible outputs included: regional capacity building, 17,000 newborns screened and habilitated, and opening of the MEHA Regional Center in Amman, Jordan in 2000. These practical achievements were seen as extremely important for maintaining active involvement of participants, for attracting and fulfilling the mandate of funders, and for building support for cross-border collaboration in the region.

**Table 3:** Lessons learned for building cross-border cooperation

**Would do the Same**
• Identify a common problem (health condition) and seize the opportunity
• Identify committed partners and bring them together
• Stay positive and keep people in the network
• Maintain symmetry in involvement of all partners
• Produce tangible and visible results
**Would do Differently**
• Build an administrative infrastructure
• Establish a firm financial basis
• Include policy makers in the network
• Keep all the partners in the loop and use their services
**Critical Events**
• Involvement of Jordanian Royal Court
Invitation in 1994 from the late King Hussein to build cross-border initiatives Prince Firas as the Patron of MEHA
• Steering Committee meetings in the region
*"Attending meetings showed that people can work together regardless of the problems in the region." (P2"*
• Opening of MEHA Regional Center in Amman, Jordan
*"It is a regional Center and a Regional NGO and it is in Jordan. It is very crucial ... seeing is believing." (P8)*
**To Ensure Future Success**
• Enhance peer recognition through research, publications and scientific meetings
• Expand the network
• Establish better human resources and organizational infrastructure
• Secure stable funding
• Educate the public about aim and accomplishments

In contrast, participants noted several things that they would do differently: ensure financial support and infrastructure early on, include policy makers, and keep everyone meaningfully involved. Viewpoints varied regarding the success of MEHA Project 1 in promoting cooperation in the region. Some saw the success as only moderate. Others pointed out that peacebuilding was quite ambitious at this point in time (second intifada), and that progress was being made in building cooperation among Israeli, Jordanian and Palestinian colleagues.

A key lesson was having broad-based involvement in the project characterized by one participant as the three Ps: *involving the policymakers, the professionals and representatives of the public at large." (P3)*

## Discussion

The Health as a Bridge for Peace framework (*WHO Resolution 34.38*, 1981) underscores an important role that health professionals can play in conflict situations [18]. However, the concept needs to be evaluated through systematic research in the field yielding evidence-based guidelines. This case study provides a relatively unique opportunity to examine and learn from a cross-border initiative during a period of intense conflict in the Middle East. Jordanian, Palestinian and Israeli health professionals were prepared to get involved in MEHA project 1 for both professional and personal reasons (Table 1). However, project implementation faced a host of complications (Table 2). Our key informants' perspectives on how these challenges were addressed provide valuable lessons and critical success factors for health and peacebuilding initiatives (Table 3). The lessons learned support the building of "cooperation networks" as a social infrastructure for underpinning health security and global health promotion.

At the same time, caution must be exercised when interpreting and applying the results from this single study. Further research is needed to evaluate and extend the findings in other health settings and sociopolitical contexts.

Also, certain decisions were made regarding the methods that warrant explanation. First, the guiding questions in the study mainly focused on having participants describe the successes and challenges, rather than inquiring directly about what attracted them. This was done to give participants room for their own stories to emerge during the interview. Second, the guiding questions were sent to the interviewees in advance to help them focus and understand the scope of the session. This may have had some steering effect, for example, in eliciting meta level reasons for involvement that included building relationships, cooperation and trust. However, these concepts were not new to study participants: i.e. they were part of early brochures describing CISEPO. The question merely opened up this aspect of CISEPO for their own perspectives to come forward as part of the discussion around successes and challenges.

Our research was conducted during 2001–2004 when increased conflict under the second intifada was taking place. However, since then broader conflicts in the Middle East and beyond (e.g. Iraq, Lebanon, Iran, Afghanistan) are having untold impact on the Israeli and Palestinian situation. This is coupled with the deep internal conflict in spring 2007 among Palestinians with Hamas taking control of Gaza and Fatah controlling the West Bank. Work under CISEPO is continuing in the region, but the level of activity has been moderated in part due to the conflict, funding restrictions for the Palestinian component, and donor fatigue (i.e. discouragement about cooperation in the region).

Critical reflection is needed to help anticipate, understand and manage both the intended and unintended consequences from a cross-border health initiative such as this study. The quotation under Challenges Faced from one participant (P9) raises a caution that good intentions could under certain circumstances result in negative consequences (e.g. 'developing an institution which might have harmful political implications'). This concern is addressed in three ways. First, colleagues associated with CISEPO are health professionals and/or leading academics who are bound by professional ethics, standards and values. They step forward to get involved through personal choice and volunteer their time. Second, oversight of activities is given by the Canadian NGO (CISEPO) and its three regional directors: Prof Ziad Abdeen, Al Quds University (Palestinian director); Prof Ziad El-Nasser, Jordan University of Science and Technology (Jordanian director); Dr Yehudah Roth, Wolfson Medical Center (Israeli director). Project 1 described in this study is also under the auspices of the *Middle East Association for Managing Hearing Loss *(*MEHA*) that CISEPO helped form. Both CISEPO and MEHA provide mechanisms for good governance and financial controls in the region. Third, activities undertaken in the region are transparent, altruistic focused and avoid any political alignment. The results from specific studies are presented at local and international conferences, and published in peer-reviewed scientific journals. This allows CISEPO/MEHA's work to be open to scrutiny by the health professional and academic communities.

A critique can be raised about the Canadian involvement in this peacebuilding initiative as 'meddling by foreigners'. In response to this concern, our approach[10] is to work together through partnerships in the region with CISEPO serving an organizing function. Emphasis is placed on equity and collaboration with all parties having a strong voice via our regional directors. In the words of one of the Palestinian participants, "we are equals in the partnership if not in our current circumstances (P4)". Indeed, the present study is an opportunity for all of our colleagues – Israeli, Palestinian, Jordanian, Canadian alike – to have an equal voice regarding the dynamics of the infant screening Project 1 as well as the successes and challenges of our cross-border activities. The role of the Canadians via CISEPO is to help organize and resource regional activities that address important health needs chosen by colleagues in the Middle East.

**Figure 2:**
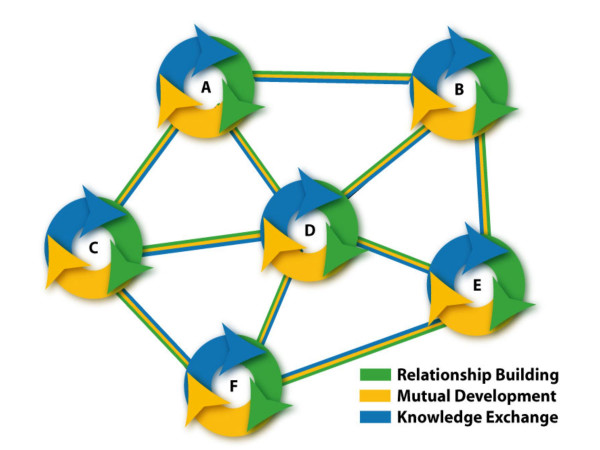
Linking projects to build a network in the region.

**Figure 3:**
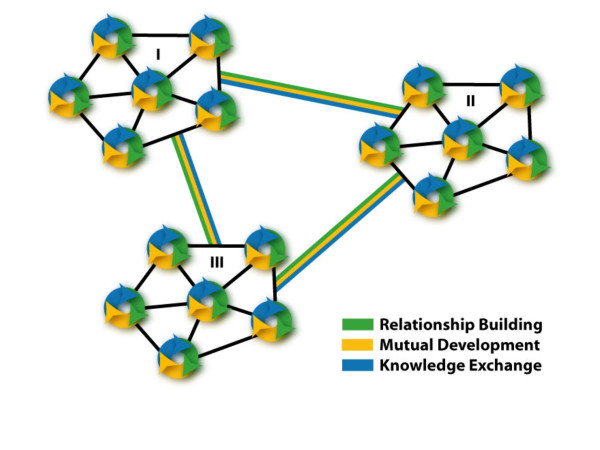
Synthesizing cooperation networks for global reach.

The findings from this study give initial support for a bi-level model (Figure 1) that integrates *project specific goals *for improving health services, clinical and population health outcomes, with *meta-level goals *for building cross-border cooperation and knowledge exchange [10]. Critical mass is created by linking projects across thematic areas (Figure 2). For example, the middle east network working with CISEPO is expanding from a focus on congenital hearing loss (A), to include activities on micro-nutrient deficiencies (B), youth health promotion including smoking prevention (C), mother/child health (D), infectious diseases (E), and continuing education through eLearning (F). A higher-level synthesis can be built across networks to expand reach and impact (Figure 3), linking with sectors beyond health such as engineering and environmental professionals collaborating on water management in the Middle East [24]. The meta level synthesis creates not only ***Knowledge Networks ***[25] but also ***Cooperation Networks ***[10,11] as social capital for peacebuilding and humanitarian aid (e.g. networks in place for facilitating Tsunami disaster relief). One can use the powerful conceptual models and measurement tools for social network analysis [26] to analyze the development and functioning of these networks.

This approach builds on the multi-level framework described by Lederach [27] that incorporates three main components: 1) integrating of short-term and long-term transformations, 2) establishing an infrastructure for peacebuilding and 3) building a peace constituency. Santa Barbara and MacQueen [7] describe an alternate framework, which include a 10 Peace through Health mechanisms, where health workers in the first five act on the health system (e.g. altruism, healing trauma) and in the second five act on the war system (e.g. dissemination of facts, mediation). The authors emphasize that peace activities can take place at multiple levels. Our approach takes an incremental, bottom-up strategy as a positive way forward during conflict. Of the 10 Peace through Health mechanisms [7] we give prominence to improving the health system through superordinate goals (e.g. collaborative capacity building) that transcend regional politics, and through humanitarian and altruistic projects that address the health needs of individuals and communities. Also, we aim to foster skills among health professionals in diplomacy and mediation that can contribute to conflict resolution at the local level.

A similar conclusion about the merits of a ground up approach was voiced by Isralowitz and colleagues[4] in their case study of training and research collaboration involving Israelis, Palestinians and international experts. Building cooperation among health professionals in conflict regions may be a small but important step. This can lay the groundwork for a trusted network of professionals and academic leaders who can mobilize humanitarian aid and rebuilding of the healthcare and public health infrastructure when a breakthrough occurs at a political level. Thus, participants in MEHA Project 1 were not only addressing an important health concern in the region but also positioning themselves to assume larger roles once the conflict subsides. According to one participant:

"What we do is we're developing a calling. And if there's any move politically, then, we have to be in there like gang busters in the health sector where we can make moves. So what we do, we're positioning ourselves." (P2)

Jameson and colleagues [28] in the second edition of their seminal work on Disease Control Priorities in Developing Countries underscore the importance of technical knowledge for improving health in low and middle income countries. As stated in their priorities compendium [29] "technical progress, in the broadest sense, works. It has been, and can be, the basis for substantial health gains, even when income growth is slow or stagnant (p.5)". However, the diffusion of technical knowledge is powerfully influenced and curtailed by social and political conflicts. This is the central thesis of Birn's [30] critique of the 14 Grand Challenges initiative funded by the Bill and Melinda Gates Foundation. Numerous examples are given where technical solutions (even 'traditional' ones such as bed nets) are hampered by the political and power structures that work against equitable distribution.

We advocate a parallel process to knowledge diffusion entailing the diffusion of cooperation to support health security and global health promotion. The central concept is the creation of 'cooperation networks' built through cross-border health initiatives. There are parallels with the Cochrane Collaboration [31] that generates systematic reviews of the effects of health care interventions. The Cochrane Collaboration comprises centers in 15 countries, 50 topic-based collaborative review groups and about 6,000 members. What we propose is an analogous process that would synthesize Cooperation Networks including best practices for peacebuilding.

Project 1 was completed in June 2004 with 17,000 Israeli, Jordanian and Palestinian newborns screened and habilitated for hearing loss. The collaboration is continuing. In 2005–2006, over 163,000 Jordanian newborns in underserved communities have been hearing tested in the recently completed *MEHA *universal screening project. On January 9, 2007, responsibility for the national program of early detection of hearing loss was transferred from MEHA to the Jordan Ministry of Health as an integral part of its offer of services to new born babies. Jordan thus became the fourth country in the world with a national program of universal hearing loss detection in new born infants.

Some other significant cross-border initiatives for CISEPO [32] include youth engagement in community health promotion projects involving Bedouin, Palestinian and Jewish grade 9 students[19], collaborative eLearning and continuing professional development[11], genetics research on hearing loss [20-23] and joint publications in international scientific journals[10,11,14,15,19-23]. Also, a large number of professionals and students have been involved. In addition to its operating network of more than 1000 individuals in Canada and the Middle East, *CISEPO *benefits from the ongoing contributions of more than 100 volunteer consultants who are senior academics and business leaders. *CISEPO *has built active partnerships with more than 20 hospitals in the Middle East (12 Israeli, 8 Jordanian, 1 Palestinian); 10 universities (5 Israeli, 4 Jordanian, 3 Palestinian); the Royal Medical Service (RMS) of Jordan; and dozens of Arab and Israeli mother and child health centres and NGOs. *CISEPO *has arranged for more than 2500 Arab and Israeli professionals and ordinary citizens to meet face-to-face in the Middle East through joint educational and research projects and public workshops.

## Conclusion

Despite many challenges, health initiatives can bring individuals together in cross-border collaboration under conditions of major conflict. These initiatives can help build mutual respect and understanding, while providing benefits to all the parties involved. According to one of our study participant, these programs bring *"kindred spirits working together" (P7) *to find solutions to common problems.

The infant screening initiative placed heavy demands on individuals during a period of heightened conflict in the region. Fostering personal relationships was critical for its success. The close personal network that evolved proved to be a source of professional, emotional and social support. An equally powerful attractor was an appreciation by participants that Project 1 was part of a broader goal for building cooperation in the region. Indeed, as echoed by participant (P6) in the opening quotation of this article, the positive impact of first meeting together can be transformative.

## Competing interests

The author(s) declare that they have no competing interests.

## Authors' contributions

Harvey Skinner designed the study, was responsible for data acquisition and interpretation, and principal writer of the paper. Abi Sriharan was co-designer of the study, contributed to data acquisition, conducted the data analyses and was a co-writer of the paper.

## Funding

This study was supported by grants from the Canadian Institutes of Health Research (CIHR), Saul A. Silverman Family Foundation (SASFF) and the Canadian International Development Agency (CIDA). These funding sources had no role in the writing of the paper.

## Ethical approval

An ethics protocol for this study was approved by the Human Subjects Ethics Review Committee, University of Toronto.
